# Endoplasmic reticulum stress triggers unfolded protein response as an antiviral strategy of teleost erythrocytes

**DOI:** 10.3389/fimmu.2024.1466870

**Published:** 2024-11-26

**Authors:** Maria Salvador-Mira, Ester Sanchez-Cordoba, Manuel Solivella, Ivan Nombela, Sara Puente-Marin, Veronica Chico, Luis Perez, Ana Joaquina Perez-Berna, Maria del Mar Ortega-Villaizan

**Affiliations:** ^1^ Instituto de Investigación, Desarrollo e Innovación en Biotecnología Sanitaria de Elche (IDiBE), Universidad Miguel Hernández (IDiBE-UMH), Elche, Spain; ^2^ MISTRAL Beamline Experiments Division, ALBA Synchrotron Light Source, Barcelona, Spain

**Keywords:** red blood cells, erythrocytes, cryo-soft X-ray tomography, rainbow trout, fish, virus, VHSV, endoplasmic reticulum stress

## Abstract

**Introduction:**

Fish nucleated red blood cells (RBCs), also known as erythrocytes, play a crucial role in maintaining immune system balance by modulating protein expression in response to various stimuli, including viral attack. This study explores the intriguing behavior of rainbow trout RBCs when faced with the viral hemorrhagic septicemia virus (VHSV), focusing on the endoplasmic reticulum (ER) stress and the unfolded protein response (UPR).

**Methods:**

Rainbow trout RBCs were Ficoll-purified and exposed to ultraviolet (UV)-inactivated VHSV or live VHSV at different multiplicities of infection (MOIs). Using cryo-soft X-ray tomography (cryo-SXT), we uncovered structural and cellular modifications in RBCs exposed to UV-inactivated VHSV. Moreover, RBCs were treated with 4-phenylbutyric acid (4-PBA), an ER stress inhibitor, to investigate its effect on viral replication. Quantitative real-time PCR was also used to analyze the expression of genes related to the UPR and other related cellular pathways.

**Results and discussion:**

Beyond their antiviral response, RBCs undergo notable intracellular changes to combat the virus. Cryo-SXT highlighted a significant increase in the ER volume. This increase is associated with ER stress and the activation of the UPR pathway. Interestingly, VHSV replication levels augmented in RBCs under ER-stress inhibition by 4-PBA treatment, suggesting that rainbow trout RBCs tune up ER stress to control viral replication. Therefore, our findings suggested the induction of ER stress and subsequent activation UPR signaling in the antiviral response of RBCs to VHSV. The results open a new line of investigation to uncover additional mechanisms that may become novel cellular targets for the development of RBC-targeted antiviral strategies.

## 1 Introduction

The main function of red blood cells (RBCs) is to transport oxygen and carbon dioxide, but these cells also play an important role in biological processes related to the immune response in fish (1, 2), such as the recognition of pathogen-associated molecular patterns (PAMPs) (2), the processing and presentation of antigens (3), and the production of effector molecules and cytokines (2, 4). Multiple studies have shown that fish RBCs are also involved in the immune response against viral infections (4–6). Understanding how fish respond to a viral infection is of utmost importance in the search for new preventive treatments, especially for the aquaculture industry.

When exposed to viral hemorrhagic septicemia virus (VHSV), fish RBCs undergo intracellular changes that prevent viral replication (5). For example, autophagy and antigen processing may be involved in minimizing the number of infectious particles in the cell (6–9). On the other hand, other fish rhabdoviruses may manipulate autophagy to evade immunity, promote replication and facilitate their release from infected cells (10–12). Transmission electron microscopy (TEM) has allowed visualization of defense mechanisms in VHSV-exposed RBCs related to the elimination of viral proteins and antigen presentation, which is the formation of vesicles similar to autophagosomes (7). The adaptive immune response of fish RBCs involves the expression of major histocompatibility complex (MHC) class I and II molecules (7, 13). The expression of these molecules implies that RBCs can present antigens and act as atypical APCs, linking RBCs to a vaccine-triggered immune response. Similar to mammalian RBCs, fish RBCs have promise as platforms for immunostimulants or vaccines given that they can express antigens encoded by a DNA vaccine and modulate the expression of interferon-related genes when expressing these antigens (14, 15).

At the intracellular level, the organization of organelles dictates the functional specificity of the cell depending on the environment. This subcellular arrangement may be key to understanding the structure-function trade-off in response to various stimuli. Nevertheless, examination of the morphological organization of a cell in its native state is hindered in part by current analytical platforms and microscopy techniques that lack high resolution and require sample sectioning (16, 17). A new approach, based on full-field cryo-soft X-ray tomography (cryo-SXT), could overcome these limitations given the potential to visualize the three-dimensional nanoscale structure of an intact cryopreserved cell. Moreover, the fast acquisition and large field of view enable rapid collection of tomographic image data. Segmenting this data into usable features is crucial for deriving biologically relevant information from cryo-SXT tomograms (18–20). Using cryo-SXT, Perez-Berna et al. (21) visualized cell structural and topological differences generated by viral exposure and noted a volumetric increase in some cells, especially in the endoplasmic reticulum (ER).

In the present work, we analyzed cryo-SXT images of rainbow trout RBCs exposed to UV-inactivated VHSV (VHSV_UV_), using the MISTRAL beamline of the ALBA Synchrotron. The purpose of the work was to investigate the structure-function link between the spatial arrangement of organelles and the cellular response after VHSV_UV_ exposure. Notably, virus inactivation by a physical agent, such as ultraviolet (UV) light, preserves virus immunogenicity while destroying infectivity. Therefore, UV-inactivated virus is capable of inducing protective responses as long as the antigen remains intact.

Cryo-SXT images facilitated the identification of RBC organelles that are the core of the response to the VHSV_UV_, featuring the ER as the main protagonist. Previous transcriptomic and proteomic analysis in RBCs has documented upregulation of molecules related to ER stress, autophagy, and antigen presentation in response to virus and DNA vaccine encoding the viral antigen (7, 14). The ER plays a crucial role in maintaining cellular homeostasis by processing and folding proteins. Alterations in the ER function, such as the accumulation of misfolded proteins or increased demand for protein folding, leads to a state known as ER stress. To resolve this imbalance, cells trigger a series of signaling pathways called the unfolded protein response (UPR) (22, 23). The UPR is controlled by three main ER sensors: inositol-requiring enzyme 1 (IRE1), protein kinase R (PKR)-like ER kinase (PERK), and activating transcription factor 6 (ATF6). Under homeostatic conditions, these transmembrane sensor proteins bind to folding chaperone glucose-regulated protein 78 (GRP78), also known as binding immunoglobulin protein (BiP). Under stress, transmembrane sensor proteins dissociate from GRP78, resulting in the activation of all three pathways (24, 25). ER stress has a major impact on infections, and pathogens can modulate ER stress to their own advantage. Regarding viruses, recent studies have shown that various viral taxa, including Orthomyxoviruses such as influenza virus and Flaviviruses like dengue virus, can significantly modulate the UPR to enhance their replication and evade host immune responses (26–28). However, recent findings suggest a protective role orchestrated by ER stress that the cell activates for its survival (29).

In this work, we investigated the possible correlation between morphological changes and the role of ER stress as a first-aid pathway to invoke an antiviral response in rainbow trout RBCs in response to an inactivated virus. Combining this new knowledge with previous findings will shed light on potential molecular targets for the design of new vaccine approaches, for which RBCs are ideal candidates.

## 2 Materials and methods

### 2.1 Animals and ethical statements

Rainbow trout (*Oncorhynchus mykiss*) individuals approximately 6-7 cm in size were acquired from a certified commercial farm (Piszolla S.L., Cimballa Fish Farm, Zaragoza, Spain). The fish supplier followed strict biosecurity protocols that include the regular monitoring of stock health and compliance with veterinary standards to ensure that the fish are free of pathogens. Fish were maintained at 14°C with a recirculating, dechlorinated water system at the Miguel Hernández University (UMH) animal house facilities. Prior to experimentation, fish were acclimatized to laboratory conditions for two weeks. The number of fish samples used is expressed by an “n” in each experiment’s figure legend.

### 2.2 Cell culture and virus

The protocol used to obtain and purify rainbow trout RBCs has been previously described (5, 30). In brief, fish were bled from the caudal vein using insulin syringes (NIPRO, Bridgewater). Collected blood samples were placed in a 2 mL eppendorf tube with RPMI-1640 medium (Dutch modification) (Gibco, Thermo Fisher Scientific) supplemented with 10% fetal bovine serum (FBS) gamma irradiated (Cultek), 1 mM pyruvate (Gibco), 2 mM L-glutamine (Gibco), 50 μg/mL gentamicin (Gibco), 2 μg/mL fungizone (Gibco), and 100 U/mL penicillin/streptomycin (Sigma-Aldrich). These samples were layered out onto 2 mL of Histopaque-1077 Ficoll gradient solution (Sigma-Aldrich), a sterile-filtered, ready-to-use solution with a density of 1.077 g/mL. Two consecutive density gradient centrifugations were carried out for purification. Then, RBCs were washed twice with RPMI 2% FBS. Finally, RBCs were cultured with RPMI 10% FBS in 25 cm^2^ flasks (Nunc Roskilde) at 14°C for 24 hours before experimentation.

The VHSV strain 07.71 (31) was purchased from the ATCC (VR-1388) and cultured in fathead minnow *Epithelioma papulosum cyprini* (EPC) cell line at 14°C as previously reported (32). VHSV is known to replicate efficiently in EPC cells with a high degree of reproducibility (33).

### 2.3 Cryo-soft X-ray tomography of RBCs exposed to VHSV_UV_

VHSV (10^8^ tissue culture infectious dose 50% [TCID_50_]/mL) was inactivated by two rounds of UV-B irradiation at 1 J/cm^2^ using a Bio-Link crosslinker BLX E312 (Vilber Lourmat, BLX-E312), following previously validated methods (9). The viral suspension was placed in a shallow multi-well culture plate, to ensure minimal liquid height to maximize UV light penetration and effectiveness. Ficoll-purified rainbow trout RBCs were exposed to VHSV_UV_ in RPMI 2% FBS, at a multiplicity of infection (MOI) of 10. RBCs unexposed to VHSV_UV_ were used as a control. At 12 hours post-exposure (hpe), RBCs were placed on top of Quantifoil holey film copper TEM grids (Au-G200F1) coated with poly-L-lysine (Merck, Germany), with fiducial gold markers (100 nm; BBI Solutions, UK) and vitrified by plunge freezing in liquid ethane in a Leica EM-CPC. We selected the 12 hpe time point based on previous studies indicating that VHSV replication decreases between 6- and 24-hpe (5). This time frame was chosen to capture a critical phase for evaluating cellular responses, while considering the viral replication dynamics.

Vitrified grids were transferred in liquid nitrogen to the cryo-correlative cooling stage (CMS196 stage; Linkam Scientific Instruments, UK) to hold samples stably at 190°C during analysis. The cryo-stage was inserted into a Zeiss Axio Scope (Carl Zeiss, Germany) epifluorescence microscope to visualize the frozen grids. Selected samples were transferred under cryogenic conditions to the MISTRAL beamline (ALBA light source) at ALBA Synchrotron (34, 35). Tomographic data were collected at 520 eV, irradiating the samples for 1-2 s per projection. 520 eV is the energy range in which water is transparent for X-rays and they are mostly absorbed by carbon, allowing the visualization of biological material. In tomographic setup, images obtained at different sample orientations are computationally combined to produce a three-dimensional image, permitting the three-dimensional representation of the sub-cellular ultrastructure of whole, intact cells. A tilt series was acquired for each cell using an angular step of 1° on a 70° angular range with a Fresnel Zone plate (FZP) with a 40 nm outermost zone width and effective pixel size of 13 nm.

Each transmission projection image of the tilt series was normalized using flat-field. This process considers the possibility of different exposure times and the slight decrease of the electron beam current during the acquisition. To increase the image quality, wiener deconvolution considering the experimental impulse response of the optical system was applied to the normalized data (36). The Naperian logarithm was used to reconstruct the linear absorption coefficient (LAC). The resulting stacks were loaded into IMOD software (RRID: SCR_003297), and individual projections were aligned to the common tilt-axis using the internal cellular structures as markers (37). Then, the aligned stacks were reconstructed with algebraic reconstruction techniques (38). The visualization, feature segmentation, and quantification of the LACs and volumes were carried out using Amira 3D software (Thermo Fisher, RRID: SCR_007353). Cellular organelles are distinguished by image contrast, which is due to differential and quantitative absorption of X-ray photons in the ‘water window’, between the absorption edges of carbon (284 eV) and oxygen (543 eV) (39). The interaction of X-rays is element-specific and is due to the relative concentration of organic material and water. We used previous knowledge on the appearance of cellular organelles under cryo-SXT (40) together with representative TEM images (7, 41–45).

The euchromatin/heterochromatin (EU/HET) ratio was calculated taking into account the absorbance of the segmented material that is the corresponding LAC for each voxel of the volume reconstructed with SXT (46). For this purpose, the nuclei were analyzed and compared in terms of volume, absorbance, and shape. As a result, a difference in density can be observed, demonstrating a bimodal distribution. This allows the selection of regions of lower absorption (i.e., light areas) and regions of higher absorption (i.e., dark areas) within each nucleus. Regions with low LAC values correspond to euchromatin regions with low chromatin condensation and more transcriptional machinery. In contrast, regions with high LAC values correspond to heterochromatin, a condensed form of DNA with less transcriptional activity (47).

### 2.4 Time course of ER stress gene expression in RBCs

Ficoll-purified RBCs from the peripheral blood of rainbow trout individuals (5 x 10^5^ cells/well) were exposed to VHSV_UV_ at MOI 10 and VHSV at MOI 1 and 10, in RPMI 2% FBS at 14°C, and incubated for 6 and 24 hours. Samples were resuspended in TRK lysis buffer (Omega Bio-Tek, Inc.) and stored for RNA extraction and quantitative real-time PCR (qPCR) analysis as previously described, and detailed below (5). For differential expression analyses, we examined a set of genes that are well-known representative components of the UPR (22, 48–50). Evaluated genes and primers used can be found in **Table 1**.

**Table 1:** Sequence information for primers and probes used for qPCR and their related role.

	Gene	Sequence^a^	Amplicon length (bp)	Reference or accession number
**UPR-associated genes**	*grp78*	**F:** CCCCAGATCGAGGTCACCTT **R:** CTTGTTGCCTGTTCCCTTGTC	81	AB196459.1
	*atf6*	**F:** GCCCCAGACCTCGACTTTG **R:** GTCCACACTCAGATCCCCATCT	87	XM_036978881.1
	*calr*	**F:** GACTGTGGCGGCGGATAT **R:** GCGAGTCTCCGTGCATAGC	70	NM_001124478.1
	*atf4*	**F:** GGATCAAGAGGTCATGGTGAAGT **R:** GCTCGAGTGTATACCCCATGGA	71	XM_021588895.1
	*chop*	**F:** TTCCTCTCCTGTCTCCTCTCTTACTAG **R:** AGAGTTGCCTCTCTTGCGTTTG	76	XM_021609132.2
	*edem1*	**F:** CGACCTGTCACCCTGTGAGA **R:** TCCGGTCACAGTTGCTATTGTT	83	XM_021609305.1
	*trap1*	**F:** ATGGTCCAGAAGTGGCATGTG **R:** GCTTCCTTGGTGCTGTAGTAGGA	72	XM_036941747.1
**Antigen presentation and vesicular transport**	*mhcI*	**F:** GACAGTCCGTCCCTCAGTGT **R:** CTGGAAGGTTCCATCATCGT	175	(126)
	*mhcII*	**F:** TGCCATGCTGATGTGCAG **R:** GTCCCTCAGCCAGGTCACT **P:** CGCCTATGACTTCTACCCCAAACAAAT	67	(127)
	*exoc1*	**F:** AGCTTATCAGAGCCGTCTTTATGAA **R:** TGGAGAAGATGTGGTGGAAGTTC	105	XM_021599419.2
	*sec13*	**F:** GCAGTGATCCAGGCACAGAA **R:** CTGGGACTAGGATAGATGGTAGAAGTG **P:** ATTCCACTCCTCCTCCTACCCCCACA	105	(14)
**Viral replication**	*N-VHSV*	**F:** GACTCAACGGGACAGGAATGA **R:** GGGCAATGCCCAAGTTGTT **P:** TGGGTTGTTCACCCAGGCCGC	69	(54)
**Endogenous control**	*ef1α*	**F:** ACCCTCCTCTTGGTCGTTTC **R:** TGATGACACCAACAGCAACA **P:** GCTGTGCGTGACATGAGGCA	63	(128)

a Forward (F), reverse (R), and probe (P) primer DNA 5’-3’ sequence.

### 2.5 4-PBA and niclosamide cytotoxicity evaluation

To examine the cytotoxicity of 4-phenylbutyric acid (4-PBA) (Sigma-Aldrich) or niclosamide (Sigma-Aldrich) on rainbow trout RBCs, propidium-iodide (PI) (Sigma-Aldrich) staining was performed to discriminate between live and dead cells using flow cytometric analysis. Briefly, RBCs were treated with 4-PBA or niclosamide according to the times and conditions used in the assays. For 4-PBA, RBCs were incubated for 24 and 72 hours at the maximum concentration used (8 mM), whereas for niclosamide, RBCs were incubated for 24 hours at 10 µM. As a positive control to determine cell death, 10 μL of 50% hydrogen peroxide (H_2_O_2_) (an apoptosis inducer) was added to untreated RBCs for 10 minutes. Afterward, cells were stained with 10 µL PI (1 mg/mL). Possible cell damage was detected by measuring the fluorescence intensity of PI-stained RBCs using a FACS Canto II flow cytometer (BD Biosciences).

### 2.6 ER stress inhibition assay

To assess the functional involvement of the ER in viral defense, we used the ER stress inhibitor 4-PBA and tested the susceptibility of cells to VHSV exposure. 4-PBA acts as a chemical chaperone whose hydrophobic regions interact with the hydrophobic segments of the unfolded protein, thereby promoting protein folding and reducing ER stress (51). To evaluate the effect of ER stress inhibition on viral replication, cells were treated with 4-PBA prior to virus exposure as previously described (52). For this purpose, Ficoll-purified rainbow trout RBCs (5 x 10^5^ cells/well) were incubated for 24 hours at 14°C with 2, 4, or 8 mM 4-PBA in RPMI 10% FBS. RBCs were washed and exposed to VHSV at MOI 1 for 6 or 72 hours in RPMI 2% FBS at 14°C. Samples were stored in TRK lysis buffer for RNA extraction and qPCR analysis.

### 2.7 ER stress inhibition effect on autophagy in RBCs exposed to VHSV

To evaluate the effect of ER stress inhibition on autophagy, in the context of viral infection, Ficoll-purified RBCs (5 x 10^5^ cells/well) were pre-treated with 8 mM of 4-PBA for 24 hours at 14°C and then exposed to VHSV at MOI 10 for 6 hours. In parallel, RBCs were exposed to VHSV for 3 hours and then incubated 24 hours with 10 µM of niclosamide, an autophagic flux blocker. Then, we analyzed the expression of ubiquitin-binding protein p62 to monitor autophagic flux as well as the N-VHSV protein to quantify viral replication, by means of flow cytometry, as explained below.

### 2.8 Flow cytometry

Flow cytometry analysis was carried out using a FACS Canto II flow cytometer (BD Biosciences). RBCs were fixed with 4% paraformaldehyde (PFA) and 0.008% glutaraldehyde diluted in RPMI medium for 45 minutes. Permeabilization of RBCs was carried out in 0.05% saponin (Sigma-Aldrich) buffer for 15 minutes. We used rabbit anti-p62/SQSTM1 antibody (www.antibodiesonline.com; Ref #ABIN2854836, RRID: AB_3096920) diluted 1/250 in permeabilization buffer to track the autophagy process. We used murine 2C9 anti-NVHSV (53) (RRID: AB_2716276) diluted 1/1000 in permeabilization buffer to track viral replication. Primary antibodies were incubated for 60 minutes. Goat anti-rabbit IgG (H+L) CF647 and goat anti-mouse IgG (H+L) CF488 (Sigma-Aldrich) diluted 1/200 in permeabilization buffer, and incubated for 30 minutes, were used as secondary antibodies. RBCs were stored in 1% PFA in PBS.

### 2.9 Analysis of genes involved in UPR and related cellular signaling pathways in RBCs from VHSV-challenged rainbow trout

Individual rainbow trout were challenged with 50 µL VHSV (10^8^ TCID_50_/mL) resuspended in RPMI 2% FBS by intramuscular injection. Negative controls were injected with 50 µL RPMI 2% FBS. Animals were kept at 14°C over the course of the infection. Fish were sacrificed at 2 days post challenge (dpc), and peripheral blood collected from the caudal vein was purified to obtain the RBCs as described above. RBCs were stored in TRK lysis buffer for RNA extraction, and gene expression was analyzed by qPCR. We analyzed the genes involved in UPR and related cellular pathways listed in **Table 1**.

### 2.10 RNA isolation, cDNA synthesis, and gene expression by quantitative real-time PCR

Samples were first digested with Proteinase K solution (Omega Bio-Tek) and then RNA was isolated using the E.Z.N.A. Total RNA Kit (Omega Bio-Tek) according to the manufacturer’s instructions. Briefly, samples were applied to the HiBind RNA Mini Column, where total RNA binds to the matrix. Cell debris and contaminants were removed by a series of wash steps. Finally, high-quality RNA was eluted in DEPC-treated water. TURBO DNase (Ambion, Thermo Fisher Scientific Inc.) was used to remove residual genomic DNA as previously described (5). RNA was quantified with a NanoDrop spectrophotometer (Nanodrop Technologies). cDNA was synthesized from RNA using M-MLV reverse transcriptase (Invitrogen, Thermo Fisher Scientific) as previously described (54). qPCR reactions were performed in a total volume of 20 μL containing cDNA, 900 nM of each primer, 10 μL of TaqMan Universal PCR master mix (Thermo Fisher Scientific) with 300 nM probe, or 10 μL of SYBR Green master mix (Thermo Fisher Scientific). qPCR was carried out using the QUANTSTUDIO 3 system (Applied Biosystems, Thermo Fisher Scientific Inc.). Cycling conditions were 50°C for 2 min and 95°C for 10 min, followed by 40 cycles at 95°C for 15 s and 60°C for 1 min (5). Gene expression was analyzed by the 2^-ΔCt^ or 2^−ΔΔCt^ method (55) as indicated in each experiment, and the *ef1α* gene was used as an endogenous control. Primers and probes sequences are listed in **Table 1**. The number of samples analyzed in each experiment is indicated by an “n” in the figure legends.

### 2.11 Confocal microscopy

To visualize the ER changes in RBCs in response to VHSV, the maximum concentration of 4-PBA (8 mM) was chosen. RBCs were incubated with 4-PBA for 24 hours prior to VHSV exposure. Then, RBCs were exposed with VHSV at MOI 1 for 6 hours. Cells were washed with fresh medium and stained with ER-Tracker Red reagent (BODIPY TR Glibenclamide, Thermo Fisher Scientific Inc.) for 30 minutes, CellMask Green Plasma Membrane Stain (Thermo Fisher Scientific Inc.) for 15 minutes, and Hoechst reagent (Thermo Fisher Scientific Inc.) for 5 minutes. Dyes were diluted 1:1000. Images were taken with a Zeiss LSM900 with Airyscan 2 and analyzed with ZEN 3.2 (Blue Edition) software (Carl Zeiss Microscopy, RRID: SCR_013672). This software was also used to measure the fluorescence intensity in the Red Channel (“ER staining”) and to represent and quantify ER volume at multi-depth along the z-axis (Z-stacks). Volume rendering was performed using IMARIS software v9.3 (Bitplane, RRID: SCR_007370).

### 2.12 Software and statistics

GraphPad Prism 6 software (GraphPad Software Inc., RRID: SCR_002798) was used for graphic creation and statistical analysis. Statistical tests and *P* values are indicated for each assay. Flowing software 2.5.1 (www.flowingsoftware.com/, RRID: SCR_015781) was used to process and analyze flow cytometry data, and Floreada.io software (RRID: SCR_025286) was applied to generate histograms and dot plots for cytotoxicity assays. ZEN 3.2 (Blue Edition) software and IMARIS v9.3 were used to analyze and produce confocal microscopy images. Amira Thermo Scientific software (Thermo Fisher) was used for the analysis and processing of images obtained by cryo-SXT.

## 3 Results

### 3.1 VHSV_UV_ induces ultrastructural alterations in RBCs

**Figure 1:**
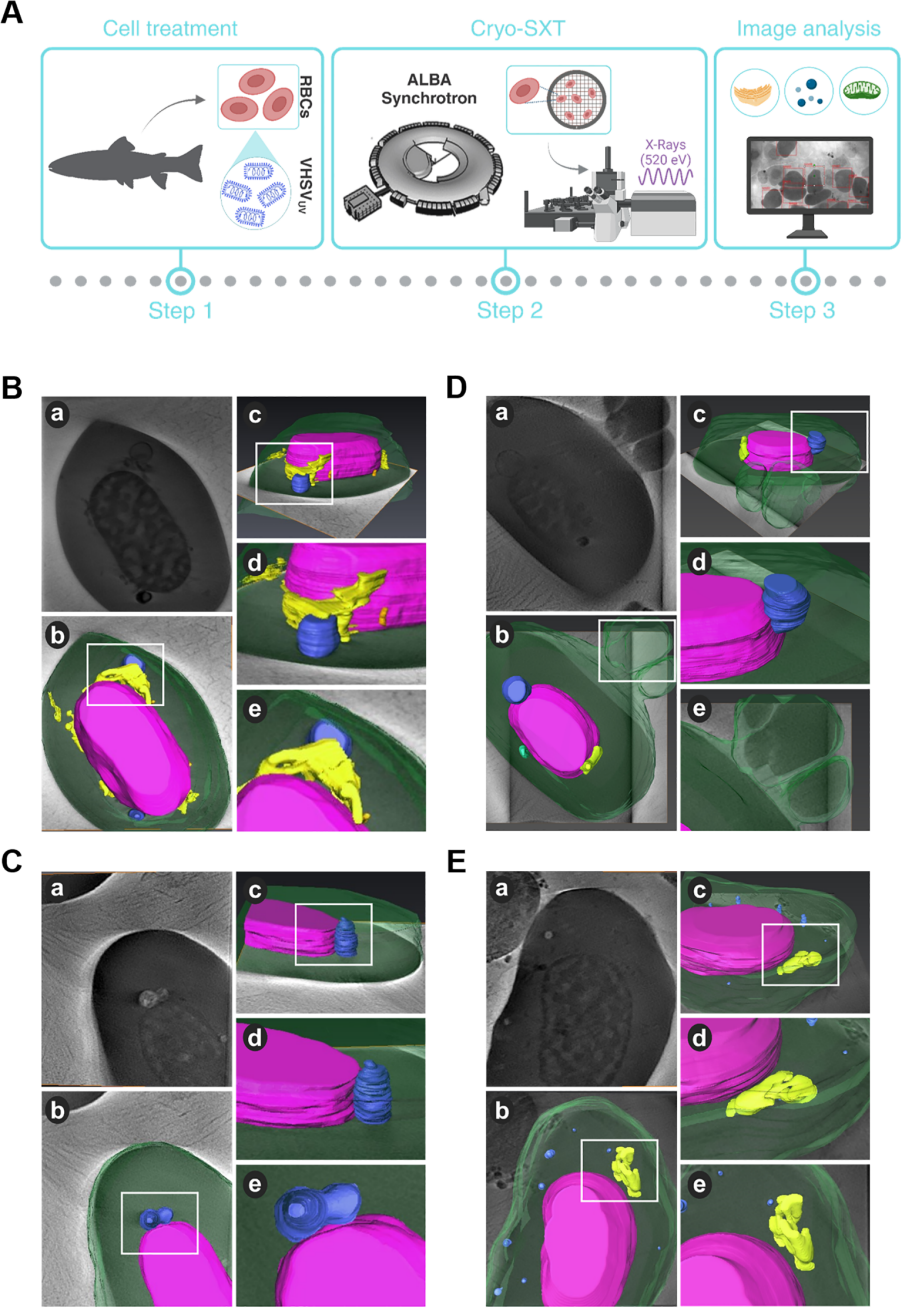
Structural and topological differences in RBCs induced by VHSV_UV_. **(A)** Workflow of experimental process. **(B–D)** Cryo-SXT images of VHSV_UV_-exposed RBCs. **(E)** Representative cryo-SXT image of control RBCs (RBCs unexposed to VHSV_UV_). Within each panel, section (a) is the cell tomography, and sections (b, c) are three-dimensional representations of the RBC in different orientations. Sections (d, e) are magnifications of the highlighted areas (white squares) of the cell. The nucleus is shown in purple, the endoplasmic reticulum in yellow, autophagosome-like vesicles in blue, mitochondria in green, and the cytoplasm and nascent EVs appear in translucent green. Volume rendering was performed with Amira software.

**Figure 2:**
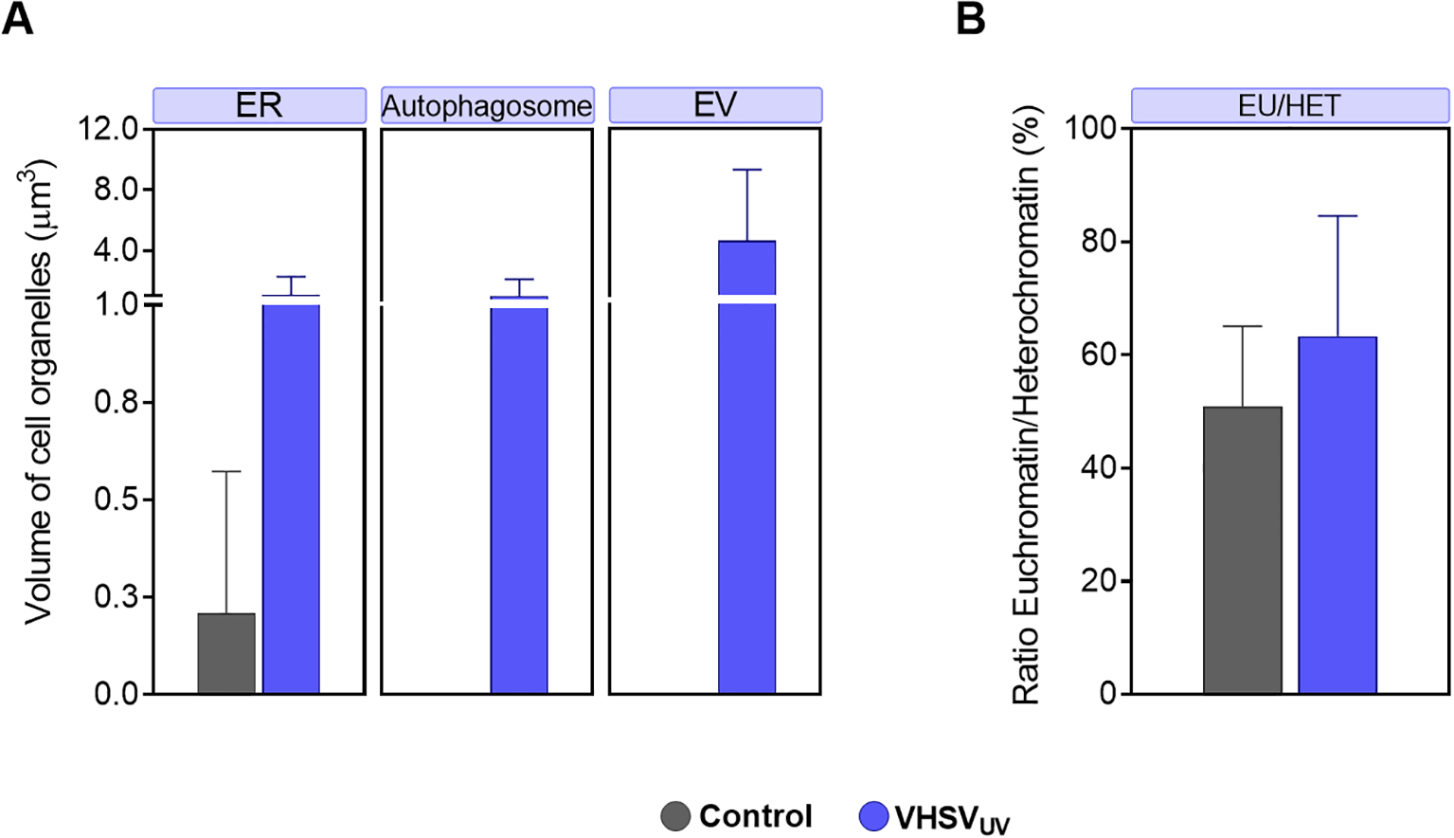
Volume quantification of ultracellular alterations in RBCs induced by VHSV_UV_. **(A)** Volume of cellular organelles of rainbow trout RBCs exposed to VHSV_UV_ in comparison to control RBCs, expressed in μm^3^. ER, endoplasmic reticulum; EV, extracellular vesicle. **(B)** Euchromatin-heterochromatin (EU/HET) ratio in VHSV_UV_-exposed rainbow trout RBCs expressed in %. Calculations were made with the Amira software. Data represent the mean ± standard deviation (n=3). A non-parametric Mann-Whitney test was applied for statistical analysis.

Three-dimensional reconstruction of cryo-SXT images of control RBCs and VHSV_UV_-exposed RBCs was carried out to gain insight into the structural alterations that occur at an intercellular level (**Figure 1**). The sample preparation and data collection steps carried out for cryo-SXT are outlined in **Figure 1A**. We also calculated the volume of the organelles of interest and the level of structural packing of the chromatin (**Figure 2**).

Based on 3D volume representations, we identified subcellular differences between VHSV_UV_-exposed and control RBCs. We have been able to clearly identify cell organelles such as ER, mitochondria, autophagosomes and extracellular vesicles (EVs). However, cryo-SXT has limited resolution visualization of some cellular structures, such as the Golgi apparatus.

Regarding ER structures, the most striking result was the thickening of the ER in VHSV_UV_-exposed RBCs (**Figure 1B**). Another distinguishing feature was the increased number and size of autophagosome-like vesicles (**Figures 1B–D**) in contrast to control RBCs (**Figure 1E**). Interestingly, these structures are often located close to the nucleus where the ER is found. Furthermore, we noted protrusions on the plasma membrane of VHSV_UV_-exposed RBCs, which could correspond to the secretion of EVs (**Figure 1D**). Volumetric quantification supported these observations: we detected higher ER volume and more autophagosome and EV formation in VHSV_UV_-exposed vs control RBCs (**Figure 2A**).

VHSV_UV_-exposed RBCs had higher EU/HET ratios than control RBCs (**Figure 2B**), suggesting increased transcriptional activity in VHSV_UV_-exposed RBCs. The selection of the LAC regions corresponding to euchromatin and heterochromatin is depicted in **Supplementary Figure 1**.

### 3.2 UPR is upregulated in RBCs in response to VHSV or VHSV_UV_

**Figure 3:**
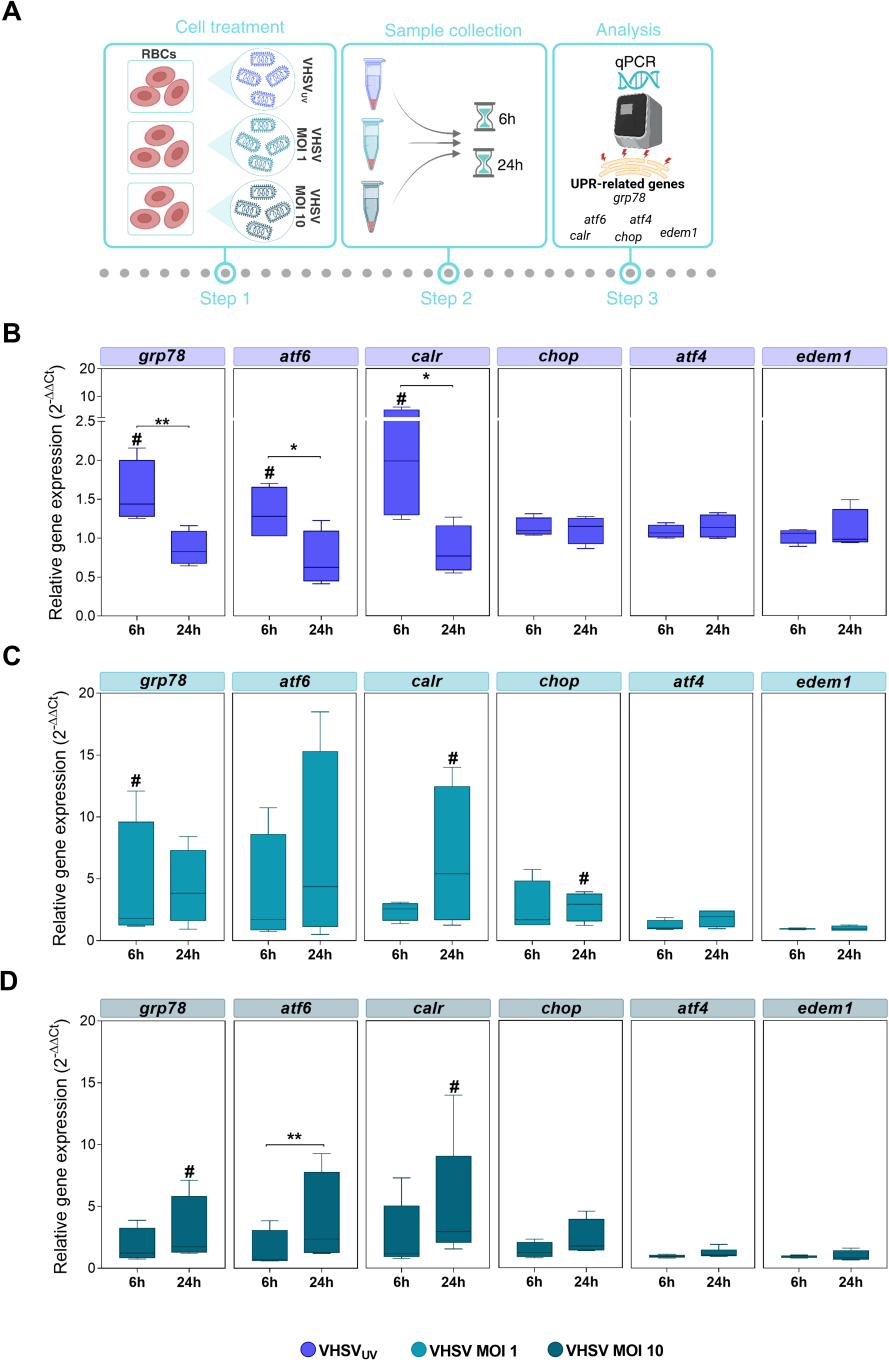
Monitoring UPR-related genes in RBCs exposed to VHSV_UV_ or VHSV, at different time points. **(A)** Workflow of experimental conditions. Box plots represent *grp78*, *atf6*, *calr*, *chop*, *atf4*, and *edem1* expression levels, in Ficoll-purified RBCs exposed to VHSV_UV_ **(B)**, VHSV MOI 1 **(C)**, and VHSV MOI 10 **(D)**, at 6 and 24 hpe. Gene expression was relativized to control cells (unexposed RBCs). *ef1α* was used as an endogenous control gene. Data represent the mean ± standard deviation (n=4). For statistical analysis, the Kruskal-Wallis test with Dunn’s multiple comparisons test was applied between each condition. #*P*<0.05 with respect to the control. *P<0.05 and **P<0.01 between time point of exposure.

We evaluated the expression of major UPR genes and their downstream targets in response to ER stress activation by VHSV_UV_ and live VHSV, at two time points. The goal was to identify a differential pattern of gene expression in the experimental conditions of interest: RBCs exposed to VHSV_UV_ (**Figure 3B**); RBCs exposed to VHSV MOI 1 and MOI 10 (**Figures 3C, D**, respectively). The experimental workflow is detailed in **Figure 3A**.

The results showed significant upregulation of the master controller of ER stress response *grp78* and ATF6 UPR branch genes, such as *atf6* and their target calreticulin (*calr*), under VHSV_UV_ or live VHSV exposure compared to control RBCs (unexposed RBCs) (**Figures 3B–D**). In detail, these three genes were overexpressed in RBCs exposed to VHSV_UV_ at 6 hpe (**Figure 3B**), compared to control RBCs, whereas in RBCs exposed to VHSV MOI 1 and MOI 10, the response was mainly delayed, with significant upregulation observed at 24 hpe (**Figures 3C, D**), except for *grp78* expression in RBCs exposed to VHSV MOI 1 at 6 hpe. Regarding PERK UPR branch, we analyzed the expression of *atf4* and *chop*. In relation to IRE1 UPR branch, we evaluated the ER degradation enhancing alpha-mannosidase like protein 1 (*edem1*) gene, which is a target gene of XBP1. Neither in RBCs exposed to VHSV_UV_ (**Figure 3B**) nor in RBCs exposed to VHSV at MOI 1 and MOI 10 (**Figures 3C, D**, respectively) was the expression of *atf4* or *edem1* affected, compared to control RBCs. No upregulation of *chop* was observed after the exposure with VHSV_UV_, compared to control RBCs, and, likewise, *chop* expression remained unchanged at both time points (**Figure 3B**). Nevertheless, *chop* overexpression was observed in RBCs in response to VHSV MOI 1 and MOI 10, compared to control RBCs, and in particular at MOI 1, at 24 hpe, when it was statistically significant (**Figures 3C, D**).

Overall, the results indicated that RBCs exposed to VHSV_UV_ or live VHSV triggered an UPR regulation. However, it should be clarified that although VHSV_UV_ confirmed rapid induction of UPR mainly through the ER chaperone GRP78 and the ATF6 pathway, the expression levels of the related genes were higher in RBCs in response to live virus (VHSV MOI 1 and MOI 10).

### 3.3 ER stress inhibition enhances viral replication in RBCs

**Figure 4:**
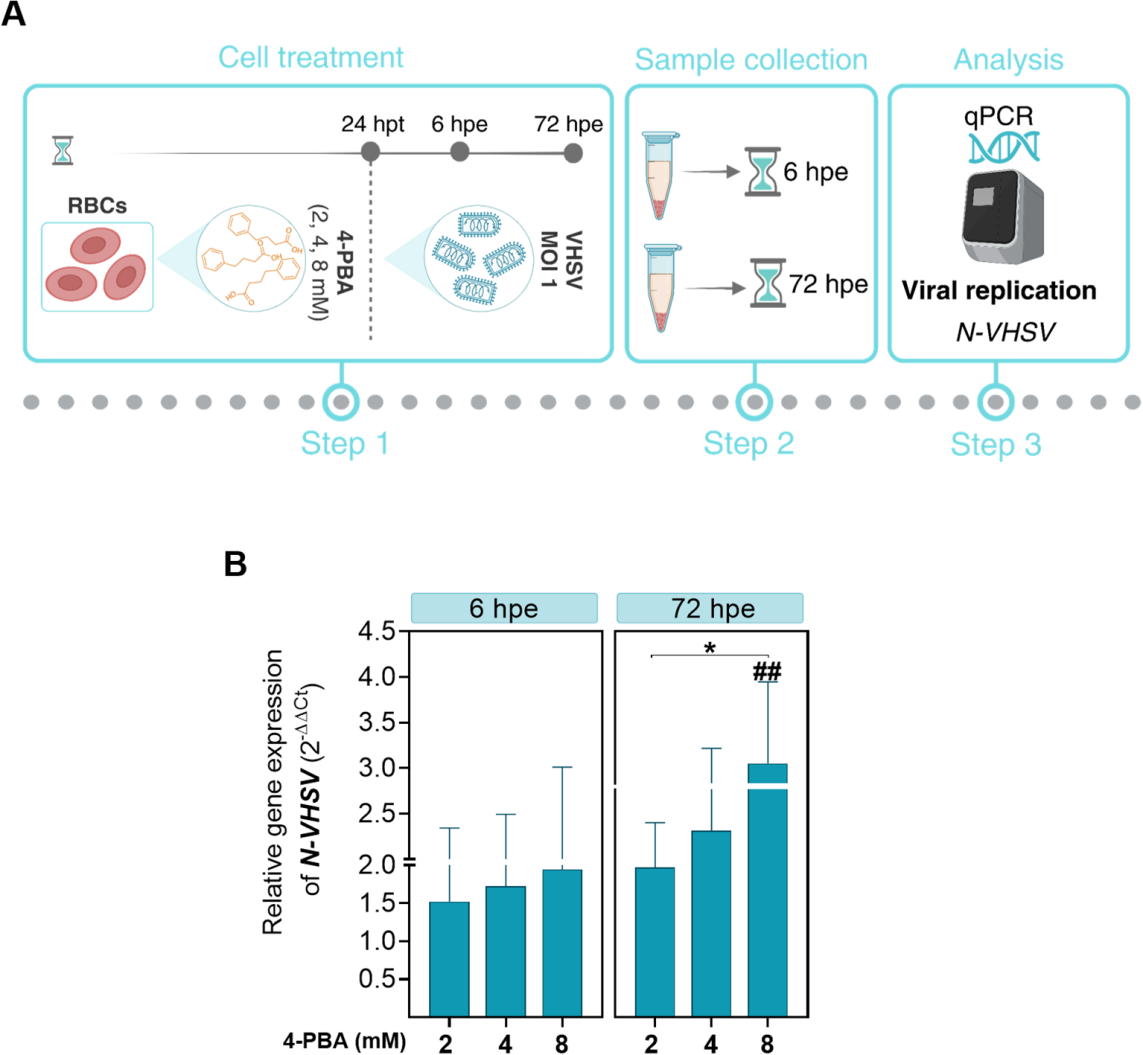
Effect of ER stress inhibition on VHSV replication in RBCs. **(A)** Workflow of experimental conditions. **(B)** *N-VHSV* gene expression in Ficoll-purified RBCs treated with 2, 4, or 8 mM 4-PBA and exposed to VHSV at MOI 1. Gene expression profile was analyzed by qPCR at 6 and 72 hpe. Gene expression was relativized to control cells (RBCs untreated with 4-PBA and exposed to VHSV). The *ef1α* gene was used as an endogenous control. Data represent mean ± standard deviation (n=6). For statistical analysis, we performed a two-way ANOVA with Tukey’s multiple comparisons test. ##*P*<0.01 with respect to the control. *P<0.05 between 4-PBA concentrations within each time point.

Pre-treatment with the ER stress inhibitor 4-PBA at different concentrations (2, 4, and 8 mM) for 24 hours was carried out prior to VHSV exposure to investigate the involvement of ER stress in the response to the virus in rainbow trout RBCs (**Figure 4A**). Then, viral replication was analyzed by qPCR of the *N-VHSV* gene in RBCs at 6 and 72 hpe. *N-VHSV* gene expression increased in a dose-dependent manner after ER stress inhibition with 4-PBA (**Figure 4B**). It should be highlighted that, as 4-PBA concentration increased, so did *N-VHSV* gene transcription, even at 72 hpe it was statistically significant for the maximum concentration used. This rise of viral replication at 72 hpe is surprising, since previous reports found that the VHSV replication cycle is halted in RBCs beyond 24 hours (5). The effect of 4-PBA on UPR modulation was also assessed in RBCs (**Supplementary Figure 2**). *grp78* and *calr* expression levels were significantly lowered in 4-PBA-treated RBCs at 6 hours, relative to their control. In contrast, at this time point, both genes were significantly upregulated in 4-PBA-treated and VHSV-exposed RBCs, when compared to 4-PBA-treated RBCs. Thus, 4-PBA appeared to attenuate UPR in RBCs while VHSV exposure acted as a stress condition that 4-PBA could not resolve. Prior to experiments, the absence of cytotoxicity of 4-PBA on RBCs was confirmed using PI staining method and measured by flow cytometry (**Supplementary Figure 3**).

**Figure 5:**
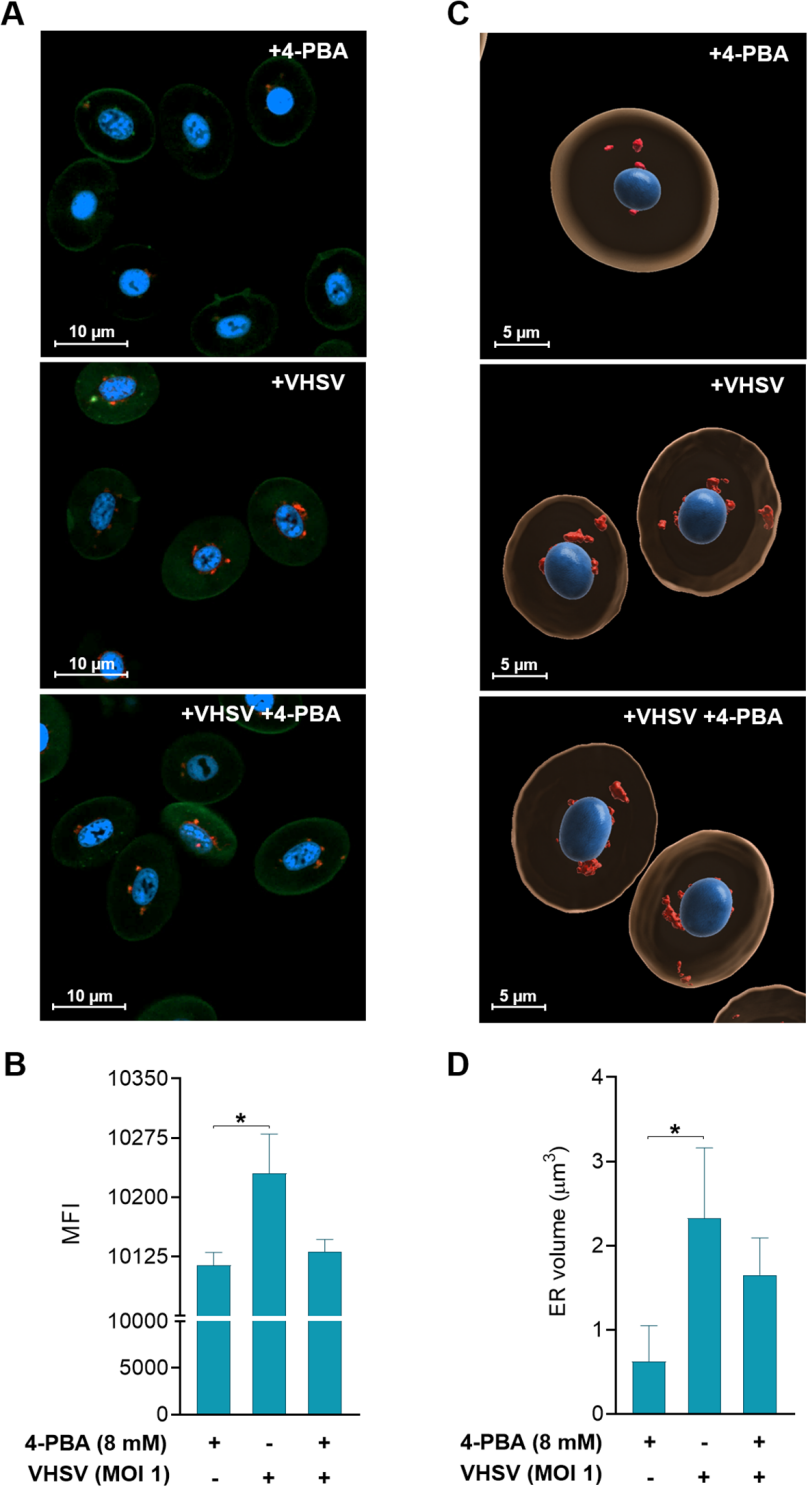
Two- and three-dimensional analysis of ER changes in response to VHSV and 4-PBA in RBCs. **(A)** Confocal laser scanning microscopy images from RBCs exposed to VHSV in the presence or absence of 4-PBA (8 mM). Images were taken at 63X magnification. The nucleus was stained with Hoechst (blue), the plasma membrane was stained with Cell Mask (green), and the ER was labeled with ER-Tracker (red). The scale bars equal 10 µm. **(B)** Mean fluorescence intensity (MFI) of the ER-Tracker signal. ZEN (Blue Edition) software was used for image processing and calculation of fluorescence intensity. Data represent the mean ± standard deviation (n=6). **(C)** Three-dimensional reconstruction of Z-stack confocal datasets. The ER is shown in red, the nucleus in blue, and the cytoplasm in beige. The scale bars equal 5 µm. **(D)** Volume quantification (µm^3^) of the ER. Data represent mean ± standard deviation (n=4). Cell volume measurement was performed with IMARIS software. For statistical analysis, the Kruskal-Wallis test with Dunn’s multiple comparisons test was used. *P<0.05.

To achieve consistency in our observations on the response of RBCs to the virus under ER stress inhibition, we performed morphological monitoring of the ER under 4-PBA treatment (8mM) and VHSV exposure. For this, we combined live-cell fluorescence microscopy through ER-Tracker with quantification of ER volume based on confocal microscopy Z-stack images. As shown in confocal microscopy images (**Figure 5A**), VHSV-exposed RBCs displayed a strong fluorescent ER signal followed by RBCs pre-treated with 4-PBA and exposed to VHSV. We calculated the mean fluorescence intensity (MFI) of the ER signal and the results showed that it significantly increased in VHSV-exposed RBCs whereas, in those RBCs treated with 4-PBA (8 mM), ER signal decreased (**Figure 5B**). Further, as shown in 3D reconstructed images from confocal microscopy, VHSV exposure led to an enlargement of the membranous network of the ER, together with ER expansion (**Figure 5C**). We next quantified ER volume in the presence or absence of 4-PBA. Analogously with MFI results, a significant increase in ER volume was visible when RBCs were exposed to VHSV but when pre-treated with 4-PBA the ER volume decreased (**Figure 5D**). It is worth pointing out here that, when considering the results of cryo-SXT, both VHSV and VHSV_UV_ shared a similar pattern in RBCs, expanding the ER in response to the stimulus.

### 3.4 ER stress inhibition effect on viral replication and autophagic flux

**Figure 6:**
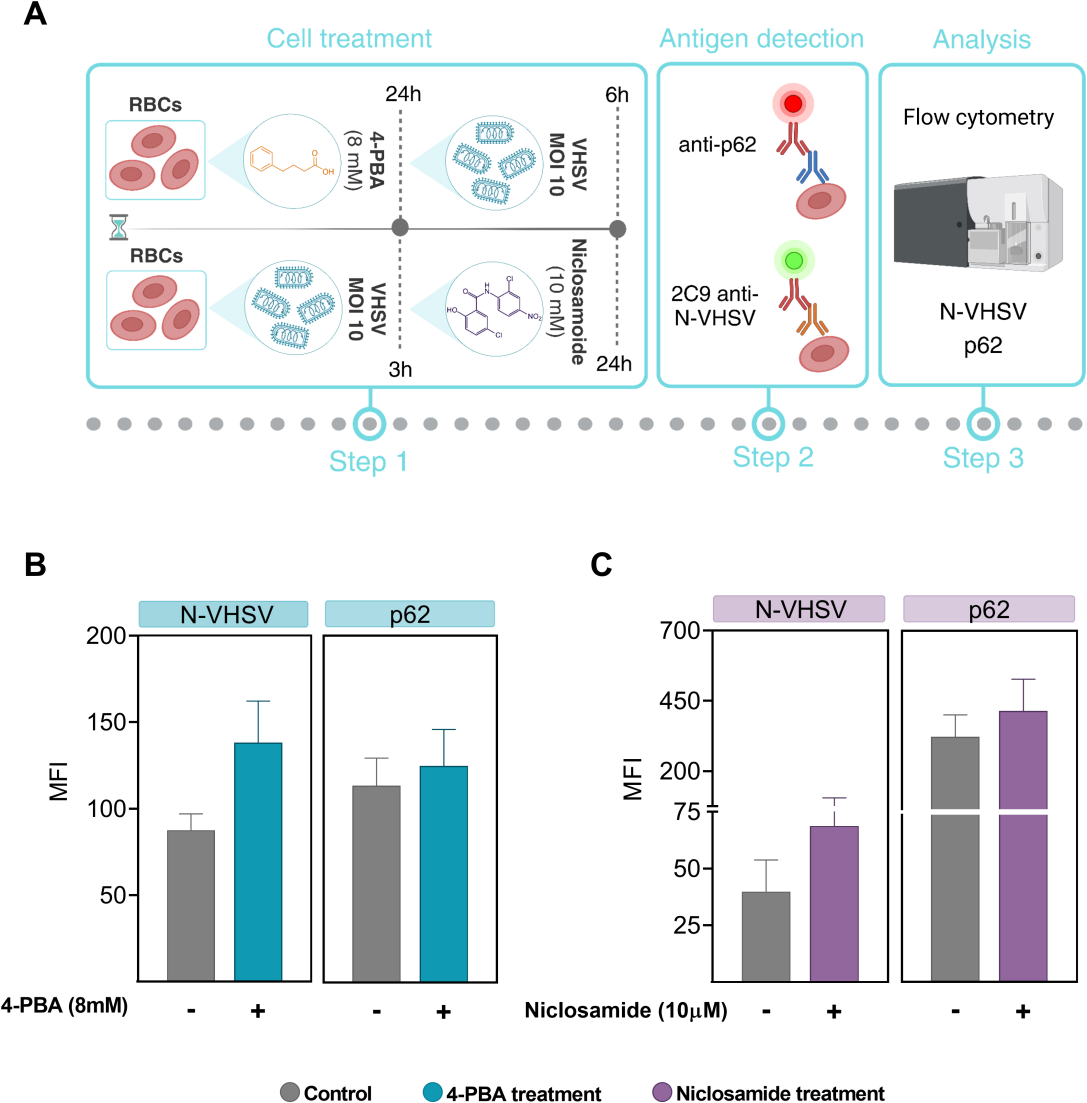
ER stress inhibition effect on viral replication and autophagic flux. **(A)** Workflow of experimental conditions. **(B)** MFI of p62 and VHSV levels in RBCs treated with 8 mM 4-PBA (blue bars) and exposed to VHSV at MOI 10, analyzed by flow cytometry at 24 hpe. **(C)** MFI of p62 and VHSV levels in VHSV-exposed RBCs at MOI 10 and treated with 10 µM niclosamide (purple bars), analyzed by flow cytometry at 24 hpe. Data represent the mean ± standard deviation (n=3). For statistical analysis, a Kruskal-Wallis test with Dunn’s multiple comparisons test was applied.

We decided to investigate whether autophagy may be altered by ER stress inhibition. For this, 4-PBA was used as an ER stress inhibitor, and niclosamide was used to block autophagic flux due to inhibition of autolysosomes formation (56). Previously, we evaluated whether niclosamide treatment had a cytotoxic effect on RBCs and results showed that niclosamide treatment at 10 µM did not induce cell damage (**Supplementary Figure 4**). As summarized in **Figure 6A**, for ER stress inhibition, RBCs were preincubated with 4-PBA and then exposed to VHSV. In the case of autophagy blockade, RBCs were first exposed to VHSV and then treated with niclosamide. By means of flow cytometry, we analyzed the ubiquitin-binding protein p62 to monitor autophagic flux as well as the N-VHSV protein to quantify viral replication under these conditions. When VHSV-exposed RBCs were treated with 4-PBA or niclosamide, we found a slight (non-significant) increase in p62 protein levels accompanied by an increase in N-VHSV protein levels, compared to untreated RBCs (**Figures 6B, C**). Although the differences were not significant, these results suggest that while both ER stress inhibition and autophagy flux blockade appear to affect VHSV replication in RBCs similarly, we cannot say whether ER stress directly affects autophagy or vice versa.

### 3.5 Transcriptomic evaluation of UPR connection with antigen presentation and vesicular transport pathways in RBCs from VHSV-challenged rainbow trout

**Figure 7:**
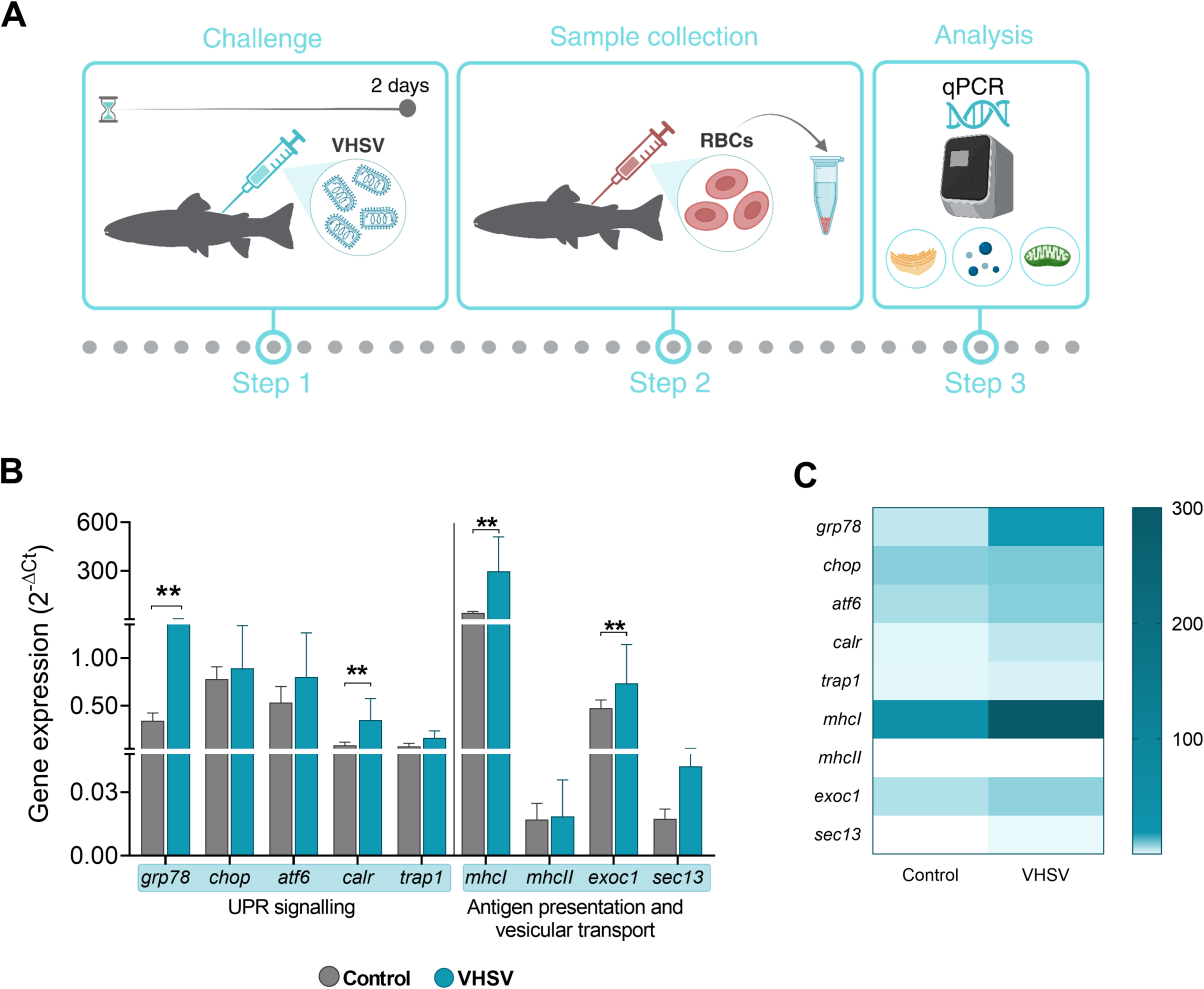
Transcriptomic profile of genes associated with UPR, antigen presentation and vesicular transport in RBCs from rainbow trout challenged with VHSV. **(A)** Workflow of experimental process. **(B)** Gene expression relativized to control RBCs (RBCs from non-challenged rainbow trout). **(C)** Heatmap showing transcription levels of selected genes. Expression levels were analyzed at 2 dpc by qPCR and normalized using *ef1α* as an endogenous gene. Data show the mean ± standard deviation (n=3). For statistical analysis, the non-parametric Mann-Whitney test was used. **P< 0.01.

We evaluated the possible correlation between the expression of UPR-associated genes *(grp78*, *chop*, *atf6*, and *calr*), and genes related to antigen presentation and vesicular transport pathways (*mhcI*, *mhcII*, exocyst complex component 1 [*exoc1*], and SEC13 homolog, nuclear pore and COPII coat complex component [*sec13*]) in RBCs of VHSV-challenged rainbow trout. Additionally, we evaluated the gene tumor necrosis factor receptor-associated protein 1 (*trap1*), which is known to be involved in ER stress by modulating the UPR and assisting in protein folding and mitochondrial stress (57, 58) (**Figure 7A**). At 2 dpc, rainbow trout RBCs showed upregulation of the genes studied, with statistically significant upregulation of *grp78*, *calr, mhcI*, and *sec13* genes (**Figure 7B**). The gene heatmap showed a marked differential ER stress, antigen presentation and vesicular transport response in RBCs from VHSV-challenged individuals (**Figure 7C**). Although we cannot definitively conclude that these pathways are directly interconnected, the observed simultaneous activation suggested a coordinated response to VHSV infection. A more comprehensive analysis is required to explore potential mechanistic links.

## 4 Discussion

Global aquaculture has faced serious economic problems due to the continuous emergence of infectious diseases precipitated by the high density of farmed fish, the geographical redistribution of aquatic species, and the international expansion of aquaculture (59). Despite efforts to develop therapeutic and prophylactic methods, currently available treatments do not meet certain essential requirements to induce protective immunity (60). Transcriptomic data of RBCs from rainbow trout individuals challenged with VHSV revealed an overrepresentation of pathways related to the ER stress, the UPR, and the signaling pathway between the ER and the nucleus (**Supplementary Figure 5**) (61). This led us to focus on the potential link between ER stress and immune response in rainbow trout RBCs, with a goal of uncovering novel methods for viral disease prevention through modulation of the UPR.

ER stress is a double-edged sword for the cell and the virus: pathogens promote their own survival and growth by selective modulation of the UPR that affects host immune responses (62–64), but activation of the UPR also exerts a protective role in the innate host defense against the invading pathogen (65, 66). A previous study detailed the link between ER stress and TLR-mediated signaling that allows cells to benefit from the protective functions of the UPR arms while avoiding CHOP-induced apoptosis (67).

In the present study, cryo-SXT revealed enlargement of the ER in RBCs exposed to VHSV_UV_. When folding capacity of ER is overwhelmed and high levels of misfolded and aggregated proteins accumulate within its lumen, ER stress occurs. This situation is characterized by the expansion or swelling of the ER lumen (68). Outstandingly, some findings have identified an altered 3D ER structure in a human hepatoma cell line during hepatitis C virus (HCV) infection (21, 69). More recently, other research reported that when rainbow trout IgM+ B cells are exposed to a bacterial lipopolysaccharide (LPS), their ER expands. This expansion is important for B cells differentiation process (70). Additionally, multiple studies have investigated the effect of viral infection on the ER (71–73). The stress induced by viral infection alters ER homeostasis, causing malfunction and accumulation of misfolded proteins (48). The UPR mechanism in the ER is activated to maintain equilibrium and promote survival through several mechanisms, including expansion of the ER membrane and the synthesis of key components in protein folding (48, 74), which leads to the enlargement of this cellular organelle observed in our results.

On top of that, tomography images of rainbow trout RBCs exposed to VHSV_UV_ showed autophagosome-like vesicles. Autophagy has been described in relation to ER stress. When proteins cannot be folded correctly, they are degraded by the ER-associated degradation complex (ERAD). If stress persists, the cell activates the autophagy pathway (72, 75, 76). Likewise, autophagosomes can originate in the ER (72), so it is not surprising that these vesicles are observed near the ER in some tomography images. The UPR and autophagy have been reported to play a role in viral pathogenesis, and in recent years there has been a great interest in the regulation of UPR and autophagy as a new therapeutic antiviral strategy (72, 77).

Since VHSV_UV_ causes ER thickening, we analyzed UPR induction as a sign of ER stress in RBCs. The key mediators of UPR, *grp78*, *atf6*, and the downstream gene *calr*, were significantly upregulated at the earliest time after exposure in RBCs treated with VHSV_UV_. Although gene activation was mild, several recent studies have shown that low levels of ER stress result in the maintenance of an adaptive UPR, which prepares the cell for subsequent stress or attack and is therefore beneficial to the cell (78–80). On the other hand, the expression of *grp78*, *atf6*, and *calr* was much more overexpressed in response to VHSV exposure, MOI 1 and 10, and mainly at the latest time after exposure. ATF6, which stimulates lipid synthesis and increases ER volume (81) was upregulated, a finding that correlated with the results of cryo-SXT. ATF6 is known as an ER stress-regulated transmembrane transcription factor that activates the transcription of ER molecular chaperones. In response to the accumulation of misfolded proteins, ATF6 translocates from the ER to the Golgi where it is processed to its active form. ER stress-induced activation of ATF6 requires a step of dissociation from the regulator GRP78, controlling its transport to the Golgi apparatus (82). Calreticulin is a multifunctional ER luminal protein, which acts as a Ca^2+^-binding chaperone (83). In addition, calreticulin is a crucial component of the UPR, and participates in protein folding and quality within the ER (84). Also, calreticulin has been connected to ATF6 branch (85). In fact, both genes were found to be simultaneously overexpressed in both VHSV_UV_- and live VHSV-exposed RBCs. GRP78 is known to function as a pivotal cellular chaperone and its regulation may promote several processes during the viral replicative cycle, demonstrating that it is highly likely that these proteins have virus- and cell-specific mechanisms of action, either antiviral or pro-viral. As a cellular antiviral mechanism, deletion of GRP78 during rotavirus infection was found to reduce infectivity by affecting the maturation of the released virion (86). In addition, some studies have reported that suppression of GRP78 increased hepatitis A virus replication in hepatocytes (87). In the same way, GRP78 can act as an intracellular antiviral factor against hepatitis B virus (88). On the other side of the coin, in the pro-viral scenario, previous reports have shown that GRP78 is positively regulated in dengue virus-infected cells and is essential for viral antigen production (89). Also, knockdown of GRP78 in human cytomegalovirus infection led to intracellular accumulation of viral particles, suggesting that GRP78 is required to accompany virion particle outgrowth (90). Additionally, burgeoning research indicated that, under unfolded protein stress, GRP78 can escape retention in the ER and translocate to the cell surface, where it has binding affinity not only for many endogenous molecules but also for those of exogenous origin, including several viral proteins (91–93). The association of GRP78 with viral proteins has been reported for hepatitis C virus, whose envelope protein E2 has been shown to tightly bind to GRP78 (94), and for coxsackievirus A9, where binding of GRP78 to MHC-I on the cell surface is essential for virus internalization (95) encephalitis virus envelope protein (96). More recently, two independent studies have shown that GRP78 can act as a co-receptor for the SARS-CoV-2 spike protein, facilitating entry into target cells (97, 98). These novel insights point to GRP78 as a multifunctional host factor and a promising line of defense or target against viral infections. Concerning PERK and IRE1 UPR branches, the expression levels of *atf4*, *chop*, and *edem1* were not affected in either VHSV_UV_- or live VHSV-exposed RBCs. Accordingly, these two UPR pathways do not seem to play a significant role in the antiviral immune response of RBCs and only the ATF6 pathway seems to be involved in this signaling.

The involvement of ER stress in the antiviral response of RBCs was evaluated at a functional level. VHSV replication levels increased in RBCs pre-treated with 4-PBA, an ER stress inhibitor, establishing a link between ER stress and the immune response triggered by RBCs against the virus. Studies have corroborated that 4-PBA treatment increases viral load in host cells (99), and other studies have shown that an ER stress inducer (e.g., 2-deoxy-d-glucose [2-DG]) inhibits viral infection (99). Similarly, tunicamycin and dithiothreitol, both inducers of ER stress, attenuated *Leishmania infantum* infection in macrophages, demonstrating a protective role of the host UPR (100). In line with previous reports, our results reveal that disruption in UPR signaling pathways by VHSV_UV_ or VHSV may generate an alarm state that helps the innate immune system to mount an adequate defense by recognizing intracellular infection (101, 102). Viral proteins, such as influenza A virus (IAV) hemagglutinin (HA) glycoproteins, have been shown to induce ER stress, resulting in HA degradation via ERAD and consequent inhibition of IAV replication (103). However, ER stress and UPR pathways have been mainly shown to be modulated in favor of viral replication and propagation. Interestingly, viruses that cause chronic infections have evolved strategies to modulate ER stress signaling, while viruses that cause acute infections use activation of these pathways as a mechanism to induce cell death (104). Rotavirus replication is most efficient under conditions of stress that disrupt host protein synthesis and cellular responses such as the UPR (104). Enterovirus 71 (EV71) induced ER stress to assist viral replication (105). Other work demonstrated that Seneca Valley virus (SVV) infection induced a proviral autophagic process in murine cells, which was regulated by ER stress, via PERK and ATF6 UPR pathways (106). Interestingly, several viral proteins such as SARS coronavirus spike protein (107), dengue 2 virus NS2B-3 protein complex (108), or human cytomegalovirus pUL38 protein (109), were identified as viral components responsible for UPR induction but not directly associated with infection.

ER functions are intrinsically related to autophagy, which is induced downstream of the UPR (110, 111) and both processes are critical for the maintenance of various aspects of cellular homeostasis. Autophagy is a cellular recycling process that can be activated by ER stress to replace unfolded proteins (112, 113). This phenomenon was observed in cryo-SXT, where we observed autophagosomes in RBCs exposed to VHSV_UV_. Autophagy is a constitutive catabolic degradation pathway that promotes cell survival under stress conditions and has a relevant role in the context of viral infections (114). For example, rainbow trout RBCs exposed to VHSV have increased autophagic activity at both the transcriptional and translational levels (7). Based on this, we investigated the link between ER stress and autophagy. For that, we monitored p62 levels in RBCs treated with an ER stress inhibitor or with an autophagic flux blocker, under VHSV exposure. We observed a slight increment in p62 levels together with an increase in viral load with 4-PBA or niclosamide treatment. These results are not conclusive but suggested that inhibition of ER stress with 4-PBA may affect autophagy, as previously reported (115).

We also observed that *in vivo* VHSV infection could also stimulate genes related to UPR, antigen presentation, and vesicular transport in RBCs. Our results indicated that these processes may cooperate in the response to the virus, as the expression of *grp78* (the main marker of the UPR), *atf6*, and *chop* increased simultaneously with the expression of *mhcI*, *mhcII*, and *calr* (antigen presentation pathways) and *sec13* and *exoc1* (which mediate vesicular transport). The expression of *trap1*, which is mainly involved in mitochondrial stress, also increased to a lesser extent. Recent reports have found that the UPR plays a role in the regulation of antigen presentation via MHC-I on dendritic cells (116), and forced expression of XBP1 improves vaccine efficacy by stimulating MHC-I/MHC-II pathways (117). Another report described how the association between mitochondria and the ER helps maintain cellular homeostasis under various pathophysiological conditions, with their interaction being mediated by mitochondrial conformational proteins and key chaperones, including calreticulin (118). Furthermore, calreticulin plays a crucial role in ER stress and is involved in the formation and presentation of MHC-I molecules on the cell surface, facilitating antigen recognition (119). This protein may act as a node between the two cellular processes, ER stress response and antigen presentation. Similarly, the secretory pathway has been linked to the UPR and vesicular transport with an active role in the regulation of ER homeostasis (120, 121). Interestingly, we observed protrusions in the cell membrane or EVs in some samples from VHSV-exposed RBCs. RBCs are the major vesicle-secreting cells in the blood, and the mechanism of vesicle biogenesis and production in RBCs has been extensively investigated in mammals (122). EVs have an important role in intercellular communication (123) via the transfer of their contents (e.g., proteins, lipids, and RNA) between cells (124). An example of this is the transfer of micro-RNA from cells infected with the Epstein-Barr virus (EBV) to uninfected cells via exosomes (125), or, in the case of rainbow trout RBCs, the production of cytokines alerting other immune cells in response to the virus (1, 5) or to a DNA vaccine that encodes the VHSV viral antigen (14).

**Figure 8:**
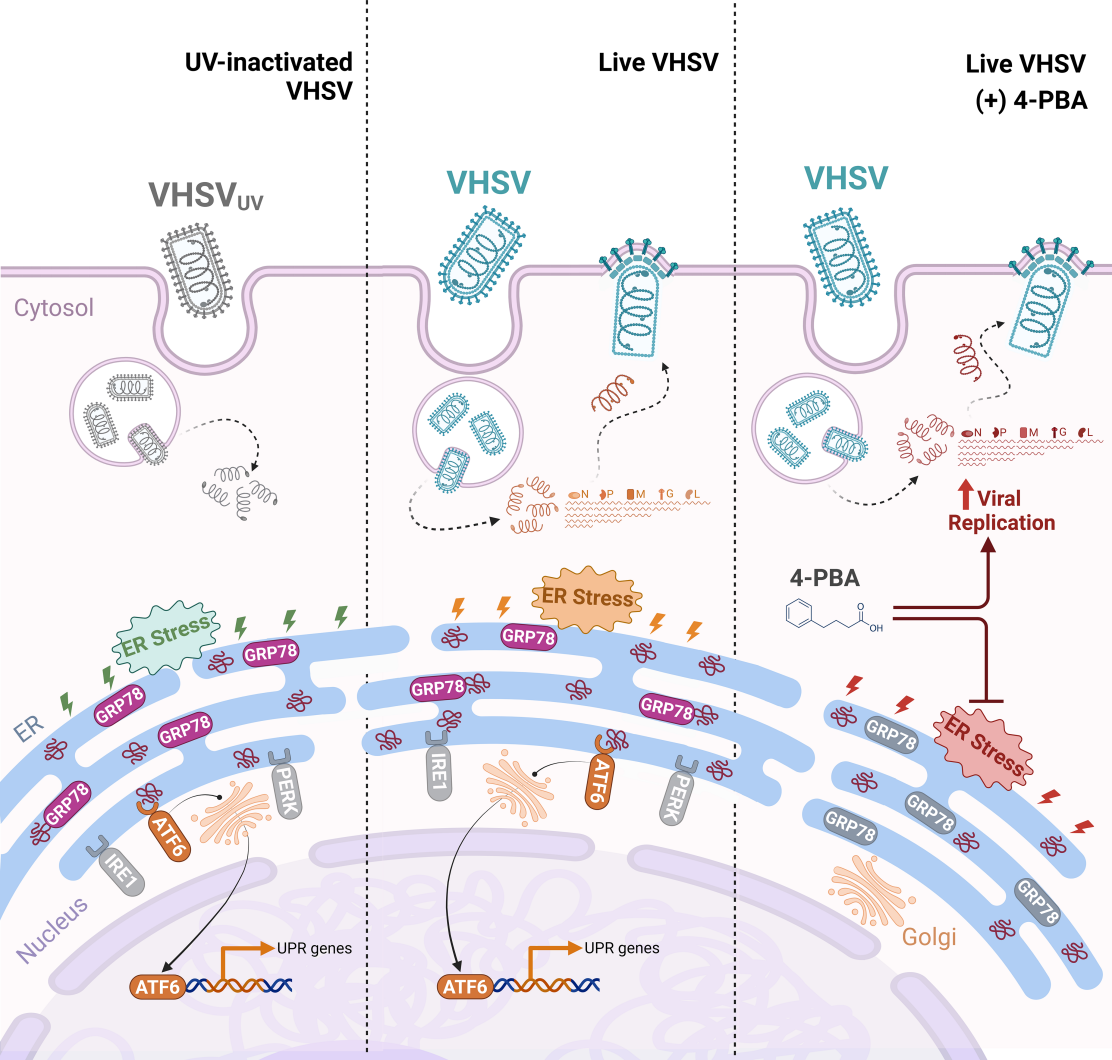
Proposed schematic illustration of the role of UPR regulation in rainbow trout RBCs antiviral response. UV-inactivated VHSV (VHSV_UV_) or live VHSV enters the RBC cytoplasm via receptor-mediated endocytosis. Then, endosome acidification leads to membrane fusion and release of the nucleoprotein (N) to the cytoplasm, containing all the components necessary for early transcription. Thereafter, ER stress is induced and the UPR is activated. (*i*) With VHSV_UV_, GRP78 is released from ER sensors and ATF6 branch is induced while PERK and IRE1 branches remain unaltered. Activation of ATF6 leads to its export to the Golgi apparatus and its translocation to the nucleus to activate the transcription of genes involved in protein folding. (*ii*) In response to live VHSV, GRP78 is released from ER sensors. ATF6 branch is activated and participates in halting viral replication. (*iii*) The inhibition of ER stress by 4-PBA is a key trigger of increased VHSV replication in RBCs.

In conclusion, our results suggested that viral-triggered ER stress contributes to raise a protective antiviral mechanism through the ATF6 UPR branch in RBCs (**Figure 8**). Moreover, the upregulation of key factors of the UPR appeared to be correlated with the induction of autophagy, antigen presentation, and vesicular transport processes. Nevertheless, it is important to stress that more detailed investigations are warranted to substantiate these findings and to clarify whether UPR signaling arises in an independent manner or is mediated by other cellular mechanisms. This study provides the basis for the identification of novel cellular targets for the development of RBC-targeted antiviral strategies.

## Data Availability

The raw data supporting the conclusions of this article will be made available by the authors, without undue reservation.
