# Supplementary material for: Endoplasmic reticulum stress triggers unfolded protein response as an antiviral strategy of teleost erythrocytes
Source: Front Immunol. 2024 Nov 26;15:1466870. doi: 10.3389/fimmu.2024.1466870 (PMC11628393; doi:10.3389/fimmu.2024.1466870)
Supplement: Supplementary file 1 [file DataSheet1.pdf]

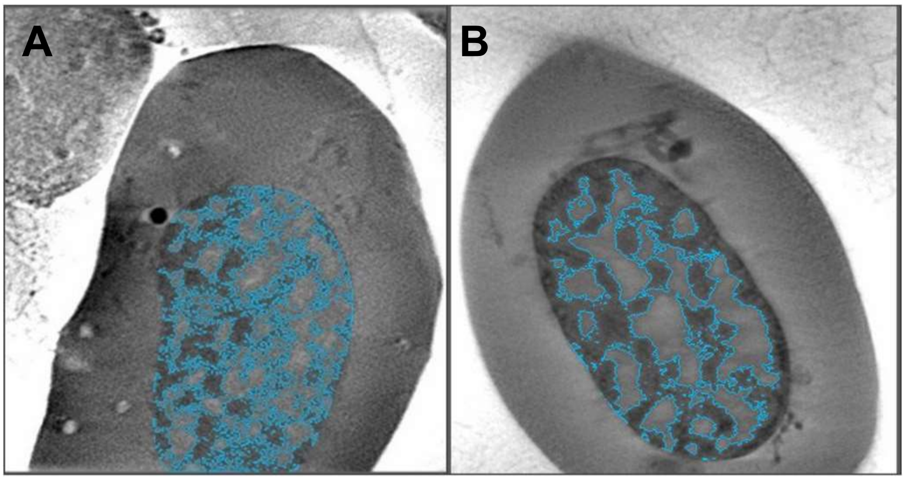

Supplementary Figure 1. Pictographic representation of euchromatin versus heterochromatin selection of (A) control RBC and (B) RBC exposed to UV-inactivated VHSV. In the tomographies, euchromatin corresponds to the regions with low LAC (linear absorption coefficient) values within the nucleus, while heterochromatin corresponds to regions with high LAC values. The resulting blue stripe selects the euchromatin and divides the two regions.
