# Supplementary material for: Endoplasmic reticulum stress triggers unfolded protein response as an antiviral strategy of teleost erythrocytes
Source: Front Immunol. 2024 Nov 26;15:1466870. doi: 10.3389/fimmu.2024.1466870 (PMC11628393; doi:10.3389/fimmu.2024.1466870)
Supplement: Supplementary file 2 [file DataSheet2.pdf]

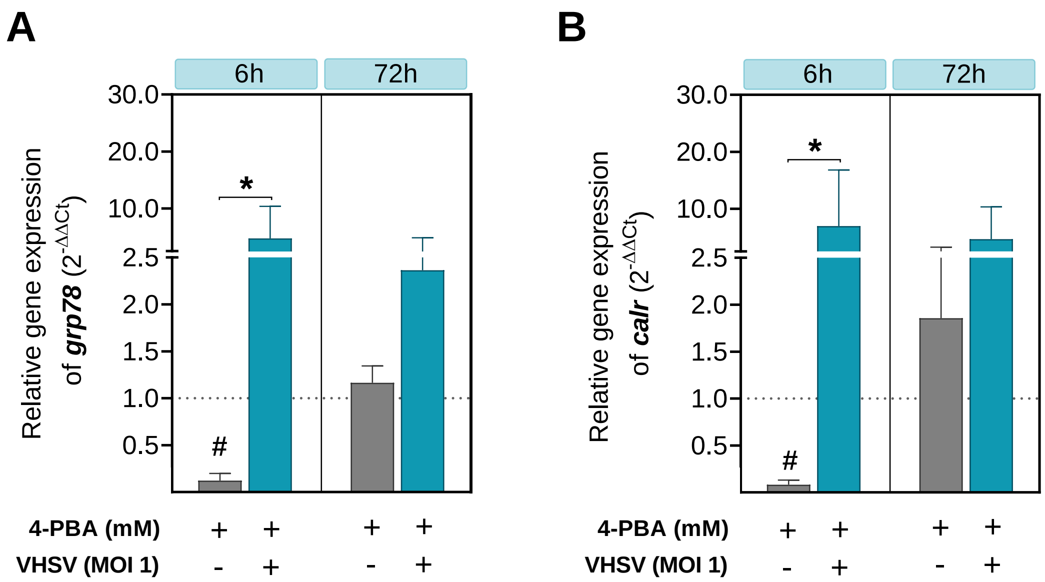

Supplementary Figure 2. Effect of 4-PBA treatment on the UPR activation in RBCs. (**A**) *grp78* and (**B**) *calr* gene expression levels in Ficoll-purified RBCs treated with 8 mM 4-PBA and/or exposed to VHSV at MOI 1. Gene expression profile was analyzed by qPCR at 6 and 72 hpe. Gene expression was relativized to control cells (untreated RBCs) (gray dotted line). The *ef1α* gene was used as an endogenous control. Grey bars represent RBCs not exposed to VHSV and blue bars refers to RBCs exposed to VHSV. Data represent mean ± standard deviation (n=4). Non-parametric Mann-Whitney test was used for statistical analysis.. #, *P*<0.01 with respect to the control. *, *P*<0.05 between VHSV-unexposed and VHSV-exposed RBCs.
