# Supplementary material for: Endoplasmic reticulum stress triggers unfolded protein response as an antiviral strategy of teleost erythrocytes
Source: Front Immunol. 2024 Nov 26;15:1466870. doi: 10.3389/fimmu.2024.1466870 (PMC11628393; doi:10.3389/fimmu.2024.1466870)
Supplement: Supplementary file 3 [file DataSheet3.pdf]

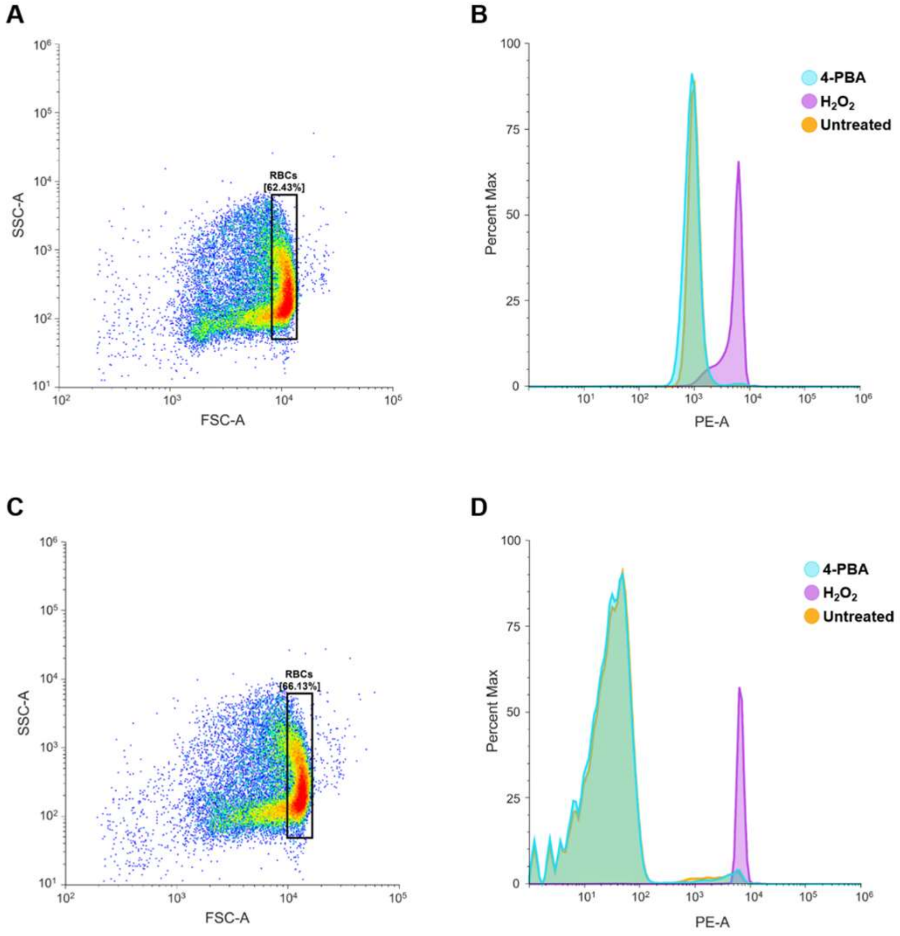

Supplementary Figure 3. Effect of 4-PBA on cell viability in rainbow trout RBCs. Cell damage was analyzed using PI-staining probe in untreated RBCs (negative control), treated with H_2O_2 (positive control), and treated with 4-PBA (8mM) for 24 and 72 hours. Representative forward (FSC) vs side (SSC) dot plots of RBCs after treatment with 4-PBA for 24 and 72 hours (**A** and **C**, respectively). Representative overlay histograms of PI-stained RBCs after 24 and 72 hours of treatment with 4-PBA (**B** and **D**, respectively) compared to untreated RBCs and RBCs treated with H_2O_2. Visualization of flow cytometry measurements was performed using Floreada.io software.
