# Supplementary material for: Endoplasmic reticulum stress triggers unfolded protein response as an antiviral strategy of teleost erythrocytes
Source: Front Immunol. 2024 Nov 26;15:1466870. doi: 10.3389/fimmu.2024.1466870 (PMC11628393; doi:10.3389/fimmu.2024.1466870)
Supplement: Supplementary file 4 [file DataSheet4.pdf]

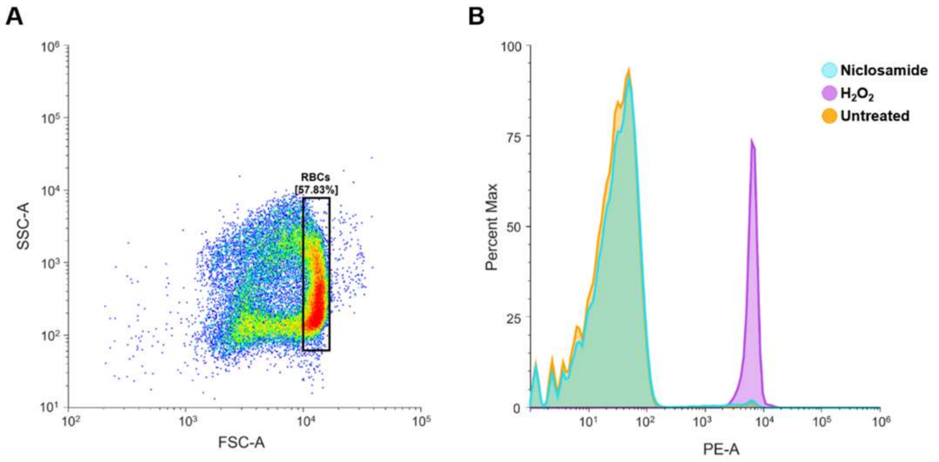

Supplementary Figure 4. Effect of niclosamide on cell viability in rainbow trout RBCs. Cell damage was analyzed using PI-staining probe in untreated RBCs (negative control), treated with H_2O_2 (positive control), and treated with niclosamide (10 μM) for 24 hours. (**A**) Representative forward (FSC) vs side (SSC) dot plot of RBCs after 24 hours of niclosamide treatment. (**B**) Representative overlay histogram of PI-stained RBCs after 24 hours of niclosamide treatment compared to untreated RBCs and treated with H_2O_2. Visualization of the flow cytometry measurements was performed using Floreada.io software.
