# Supplementary material for: Endoplasmic reticulum stress triggers unfolded protein response as an antiviral strategy of teleost erythrocytes
Source: Front Immunol. 2024 Nov 26;15:1466870. doi: 10.3389/fimmu.2024.1466870 (PMC11628393; doi:10.3389/fimmu.2024.1466870)
Supplement: Supplementary file 5 [file DataSheet5.pdf]

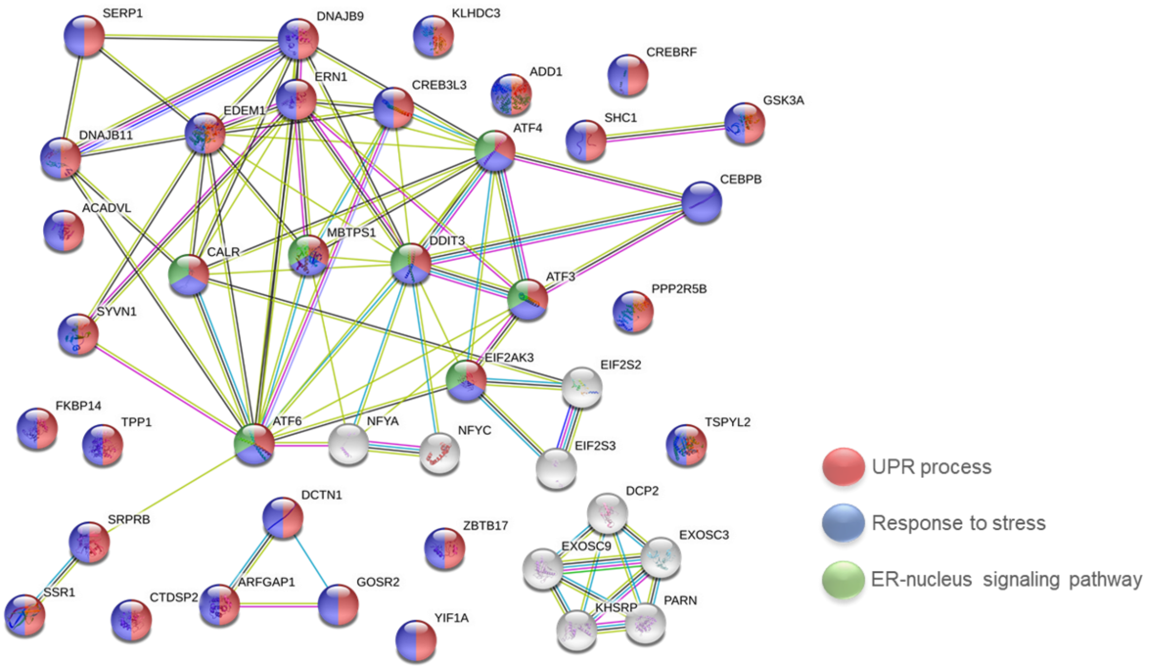

Supplementary Figure 5. The protein–protein interaction (PPI) network of RBCs from rainbow trout challenged with VHSV. Nodes represent proteins, and edges denote the interactions between 2 proteins. Node colors indicate proteins functionally annotated with STRING software (RRID:SCR_005223). The PPI enrichment *P*< 1.0-15. Data from Nombela et al. [61].
